# Pioglitazone for the Treatment of Metabolic-Associated Fatty Liver Disease in People Living With HIV and Prediabetes

**DOI:** 10.7759/cureus.19046

**Published:** 2021-10-25

**Authors:** Sarunporn Kamolvisit, Supphamat Chirnaksorn, Hataikarn Nimitphong, Somnuek Sungkanuparph

**Affiliations:** 1 Department of Medicine, Faculty of Medicine Ramathibodi Hospital, Mahidol University, Bangkok, THA; 2 Chakri Naruebodindra Medical Institute, Faculty of Medicine Ramathibodi Hospital, Mahidol University, Samut Prakan, THA

**Keywords:** human immunodeficiency virus (hiv)-positive, human immunodeficiency virus (hiv) cure, metabolic-associated fatty liver disease (mafld), pioglitazone, prediabetes

## Abstract

Background

Metabolic-associated fatty liver disease (MAFLD) is increasingly common among people living with the human immunodeficiency virus (PLHIV) and can progress to cirrhosis and cirrhotic-related complications. Pioglitazone is known to improve insulin sensitivity that results in decreasing serum fatty acids and resolution of non-alcoholic steatohepatitis. This study was aimed to evaluate the efficacy of pioglitazone for the treatment of MAFLD in PLHIV and prediabetes.

Methods

A randomized controlled trial was conducted in HIV-positive individuals with prediabetes who had evidence of a fatty liver by abdominal ultrasonography or controlled attenuation parameter (CAP) ≥ 238 decibels per meter (dB/m) through using transient elastography. Participants were randomized to take pioglitazone, 30 mg/day, (pioglitazone group) or placebo (control group) and were followed up and assessed for 48 weeks.

Results

A total of 98 participants were enrolled, 49 in each group. The mean age was 50.8 years and 66.3% were males. All participants had received antiretroviral therapy with undetectable HIV ribonucleic acid (RNA) and the mean CD4 cell count was 463.2 cells/mm^3^. The mean baseline CAP and liver stiffness were 285.7 dB/m and 5.4 kilopascals (kPa), respectively. At 24 weeks, the mean change of the CAP level was -25.7 dB/m in the pioglitazone group and -5.6 dB/m in the control group (*p *= 0.040); the mean change of liver stiffness was 0.014 kPa in the pioglitazone group and 0.403 kPa in the control group (*p *= 0.199). At 48 weeks, the mean change of the CAP level was -23.5 dB/m in the pioglitazone group and 10.2 dB/m in the control group (*p *< 0.001); the mean change of liver stiffness was -0.184 kPa in the pioglitazone group and 0.554 kPa in the control group (*p *= 0.016). The mean changes of fasting plasma glucose (FPG) at 24 and 48 weeks were -14.9 and -17.5 mg/dL in the pioglitazone group, respectively, and -3.6 and 4.5 mg/dL in the control group, respectively (*p *< 0.05). The mean change of the body mass index, lipid profiles, and liver enzymes were not different between the two groups at both time points (*p *> 0.05). No serious adverse effects were observed in either group.

Conclusions

Pioglitazone significantly reduces CAP, liver stiffness, and FPG in PLHIV with prediabetes and MAFLD. Further studies with long-term follow-up duration are warranted to determine the role of pioglitazone for clinical use in this population.

## Introduction

HIV infection continues to be a major health problem globally, with an estimated 37.7 million people living with the human immunodeficiency virus (PLHIV) at the end of 2020 [1]. With significant reductions in morbidity and mortality from antiretroviral therapy (ART), HIV infection has become a manageable chronic health condition. However, PLHIV on ART has a greater burden of chronic diseases. Large-scale cohort studies have demonstrated that non-acquired immune deficiency syndrome (AIDS) cancer, cardiovascular disease, liver disease, and chronic kidney disease are the most common causes of non-AIDS-related deaths [2-3]. The risk of liver disease is continuously elevated among PLHIV, even without prior viral hepatitis. Hepatocellular cancer, a major form of non-AIDS cancer among PLHIV, occurs among those with pre-existing liver fibrosis or cirrhosis [3].

Metabolic-associated fatty liver disease (MAFLD) is a disease spectrum ranging from steatosis to non-alcoholic steatohepatitis and end-stage liver disease [4]. While MAFLD affects approximately 25% of the general population, 20% - 63% of PLHIV have MAFLD and 14% - 63% have non-alcoholic steatohepatitis with fibrosis [5-8]. Additionally, MAFLD is an independent risk factor for cardiovascular disease, diabetes mellitus, and all-cause mortality [7].

Currently, non-invasive tools (transient elastography (TE) with controlled attenuation parameter (CAP) and liver ultrasonography) can be used as screening tools for MAFLD in PLHIV [9-11]. Clinical studies have reported a strong correlation between CAP and fat accumulation. CAP has been reported to be useful in the diagnosis of liver steatosis [9, 12]. Furthermore, TE can be used to evaluate the staging of liver fibrosis. In an area with a high burden of HIV infection, particularly in resource-limited settings, these non-invasive tools allow more PLHIV to be evaluated for MAFLD and identified for optimal interventions.

Lifestyle intervention remains the foundation of management for MAFLD. However, a previous study found that nearly half of PLHIV with MAFLD by CAP were non-obese and suggested that “lean MAFLD” may play a larger role in PLHIV than in the general population [7]. Currently, several agents have been tested but no single agent has been approved to treat biopsy-proven MAFLD.

Glitazones, agonists of peroxisome proliferator-activated receptor-gamma (PPARγ) nuclear receptors, are approved for glycemic control in patients with type 2 diabetes. They have been demonstrated to reduce insulin resistance in the liver, muscle, and adipose tissue [13]. Pioglitazone is the only drug in this group available in the market for over a decade. The ability of pioglitazone in improving insulin sensitivity has resulted in decreased blood glucose, serum fatty acids, and the resolution of non-alcoholic steatohepatitis in patients with type 2 diabetes or prediabetes [14-16], as well as nondiabetic patients [17]. Several trials among HIV-uninfected persons with non-alcoholic steatohepatitis demonstrated an improvement in liver fibrosis [14-16]. Nonetheless, there is limited data regarding this effect in PLHIV and prediabetes. Therefore, we conducted a randomized placebo-controlled trial with the primary objective to determine the efficacy of pioglitazone for the improvement of liver steatosis. The secondary objectives were to evaluate the effects of pioglitazone for the improvement of liver stiffness, lipid levels (including total cholesterol (TC), low-density lipoprotein cholesterol (LDL), high-density lipoprotein cholesterol (HDL) and triglycerides (TG), fasting plasma glucose (FPG), and body weight), as well as adverse events, between the two groups.

## Materials and methods

Study design 

A randomized placebo-controlled study was conducted among HIV-positive individuals with prediabetes and MAFLD. Patients were enrolled between June and November of 2019 and followed up for 48 weeks at the Infectious Disease Clinic, Ramathibodi Hospital, Bangkok, Thailand. Inclusion criteria were HIV-positive individuals who were 35 to 65 years of age and met all of the four following conditions: (1) prediabetes (according to the criteria of American Diabetes Association (ADA), defined by impaired fasting glucose (IFG) (FPG: 100 mg/dL (5.6 mmol/L) to 125 mg/dL (6.9 mmol/L)), or impaired glucose tolerance (IGT) (two-hour plasma glucose (2h-PG) after a 75-gram oral glucose tolerance test: 140 mg/dL (7.8 mmol/L) to 199 mg/dL (11.0 mmol/L)), or glycosylated hemoglobin (HbA1c): 5.7% to 6.4%) [18]; (2) MAFLD which was diagnosed by a fatty liver described in liver ultrasonography or TE with a CAP value ≥ 238 dB/m; (3) having at least one of the following conditions for prediabetes treatment recommended by ADA: hypertension (sitting blood pressure > 135/85 mmHg); low HDL cholesterol (< 40 mg/dL in females, < 35 mg/dL in males); elevated TG (≥ 150 mg/dL); a family history of diabetes in a first-degree relative, or a history of gestational diabetes mellitus (GDM) [19]; and (4) able to sign an informed consent.

Patients were excluded if they were/had any of the following: the presence of hepatitis virus co-infection, critical illness, or active opportunistic infection; active alcohol consumption (male > 30 g/day, female > 20 g/day), were pregnant or breastfeeding, contraindication for pioglitazone therapy (such as a drug allergy), osteoporotic fractures, or congestive heart failure. All patients gave written consent before any study-related procedures. The study was reviewed and ethically approved by the Committee of Human Rights Related to Research Involving Human Subjects, Faculty of Medicine Ramathibodi Hospital, Mahidol University (approval number MURA2018/808).

Definition of fatty liver disease

At baseline, TE with CAP and liver ultrasonography were performed on the same day and after six-hour fasting. The probe transducer was placed on the skin between the ribs at the level of the right lobe of the liver by a single experienced operator who performed 10 valid acquisitions with an interquartile range/median (IQR/M) of liver stiffness less than 0.3 [20-21]. The software then calculated the median values of liver stiffness and CAP. MAFLD was defined when CAP was ≥ 238 dB/m [22]. Ultrasonography was then performed, and the result was interpreted by a single experienced hepatobiliary radiologist. Fatty liver was identified when liver echogenicity exceeded that of renal cortex and spleen, and there was attenuation of the ultrasound wave, loss of definition of the diaphragm, and poor delineation of the intrahepatic architecture [10].

Intervention and assessment measures 

All the enrolled patients were randomly assigned (1:1) to use pioglitazone, 30 mg once daily, (pioglitazone group) or a placebo once daily (control group). All patients were advised for diet control, decreasing caloric intake by at least 30% or by approximately 500 - 1,000 kcal/day, and had at least 150 minutes/week of moderate-intensity exercise, such as brisk walking (at least 4 km/hr), gardening, tennis, or biking (slower than 16 km/hr), or an increase in their activity level by vigorous aerobic activity exercise, such as hiking uphill, running, swimming laps, or cycling (faster than 16 km/hr), for more than 60 minutes/week and prospectively followed up for 48 weeks [23-24].

After recruitment, all patients were arranged to follow-up every 12 weeks until completing 48 weeks. Patients were assessed for body weight, height, body mass index (BMI), and blood pressure. Blood samples were obtained for the evaluation of FPG, HbA1c, lipid profiles (including TC, LDL cholesterol, HDL cholesterol, and TG), complete blood count, liver function test, and creatinine at baseline, 12 weeks, 24 weeks, 36 weeks, and 48 weeks. A 75-gram oral glucose tolerance test, HIV viral load, CD4 cell count tests, and TE with CAP were performed at 24 and 48 weeks. The presence and severity of all adverse events and the compliance to treatment were also evaluated in each visit.

Statistical analysis

We calculated the sample size for each group using the n4Studies program, version 1.4.0 (Chetta Ngamjarus, Khon Kaen University, Khon Kaen, Thailand). The proportion of responded patients to calculate the sample size was derived from a previous major clinical trial [13]. We needed 98 participants, 49 in each treatment group, to achieve 80% power of the study, and with a level of significance of 0.05.

Mean changes from baseline of body weight, BMI, FPG, 2h-PG, HbA1c, complete blood count, liver function test, and lipid levels were analyzed using values from initial to the 24-week and 48-week visits.

Data were summarized as means ± standard deviation (SD) for continuous variables. The Kolmogorov-Smirnov test was used to evaluate the normal distribution of the continuous variables. A two-sample t-test was used to compare the mean values of continuous variables between the pioglitazone and control groups. The Chi-square test was used to compare categorical variables between the two groups. The mean changes in laboratory results between the pioglitazone and control groups were tested by two-sample t-tests. Non-parametric methods were used for non-normally distributed values. The statistical analyses were performed using Statistical Product and Service Solutions (SPSS), version 17 (IBM SPSS Statistics for Windows, Chicago, IL, USA). All tests were pre-specified to the 5% significance level.

## Results

A total of 98 patients were enrolled and analyzed in this study. There were 49 patients in each group. Of all, 65 patients (66.3%) were male and the mean age was 50.8 ± 6.8 years. The mean duration of known HIV infection was 10.2 ± 3.7 years. All participants had received ART with undetectable HIV ribonucleic acid (RNA) and the mean CD4 cell count was 463.2 ± 148.2 cells/mm^3^. Regarding the antiretroviral regimens, 86 patients (87.8%) had received non-nucleoside reverse transcriptase inhibitor (NNRTI)-based regimens; the others were on protease inhibitor (PI)-based regimens. There were no differences in antiretroviral regimens between the two groups. At baseline, the mean CAP and liver stiffness were 285.7 ± 43.1 dB/m and 5.4 ± 1.5 kPa, respectively. All baseline demographic data and characteristics, including age, sex, BMI, CD4, CAP, liver stiffness, lipid profiles, FPG, 2h-PG, HbA1c, and liver enzymes, were similar between the two groups (p > 0.05), as summarized in Table 1.

**Table 1:** Baseline Characteristics Between Participants in Pioglitazone and Control Groups CAP: controlled attenuation parameter; FPG: fasting plasma glucose; HbA1c: glycosylated hemoglobin; HDL: high-density lipoprotein cholesterol; HIV: human immunodeficiency virus; LDL: low-density lipoprotein cholesterol; OGTT: oral glucose tolerance test; RNA: ribonucleic acid; SD: standard deviation; 2h-PG: two-hour plasma glucose

Characteristics	Pioglitazone group (N = 49)	Control group (N = 49)
Gender, number (%)		
Male	31 (63.3)	34 (69.4)
Female	18 (36.7)	15 (30.6)
Age, years, mean ± SD	50.5 ± 6.7	51.2 ± 6.8
Body weight, kg, mean ± SD	68.8 ± 10.4	67.9 ± 10.9
Body mass index, kg/M^2^, mean ± SD	25.3 ± 3.5	24.9 ± 3.7
Family history of diabetes, number (%)	13 (26.5)	15 (30.6)
Hypertension, number (%)	14 (28.6)	14 (28.6)
Low HDL cholesterol, number (%)	8 (16.3)	9 (18.4)
Elevated triglycerides, number (%)	27 (55.1)	23 (46.9)
Fatty liver by ultrasound, number (%)		
No fatty liver	7 (14.3)	5 (10.2)
Mild fatty liver	21 (42.9)	28 (57.1)
Moderate fatty liver	20 (40.8)	16 (32.7)
Cirrhotic change	1 (2.0)	0 (0)
Transient elastography		
CAP, dB/m, mean ± SD	293.3 ± 39.7	278.7 ± 47.8
Stiffness, kPa, mean ± SD	5.7 ± 1.5	5.2 ± 1.5
Liver stiffness, number (%)		
F0 - F1 (0 - 7 kPa)	45 (91.9)	46 (93.9)
F2 (7 - 8 kPa)	2 (4.1)	1 (2.0)
F3 (8 - 10 kPa)	1 (2.0)	2 (4.1)
F4 (> 10 kPa)	1 (2.0)	0 (0)
Aspartate transaminase, U/L, mean ± SD	32.4 ± 10.5	33.4 ± 19.6
Alanine transaminase, U/L, mean ± SD	37.4 ± 16.7	41.1 ± 21.9
FPG, mg/dL, mean ± SD	106.5 ± 12.4	104.8 ± 11.1
2-hr PG after 75-gm OGTT, mg/dL, mean ± SD	134.0 ± 42.7	142.3 ± 43.4
HbA1c, %, mean ± SD	5.7 ± 0.3	5.8 ± 0.4
Lipid levels, mg/dL, mean ± SD		
Total cholesterol	208.4 ± 38.5	201.7 ± 42.4
LDL cholesterol	128.3 ± 34.3	123.7 ± 27.8
HDL cholesterol	45.2 ± 7.7	43.8 ± 9.2
Triglycerides	179.2 ± 73.20	175.6 ± 82.3
CD4 cell counts, cells/mm^3^, mean ± SD	451.1 ± 161.1	475.1 ± 135.6
HIV RNA < 40 copies/mL, number (%)	49 (100)	49 (100)

**Figure 1:**
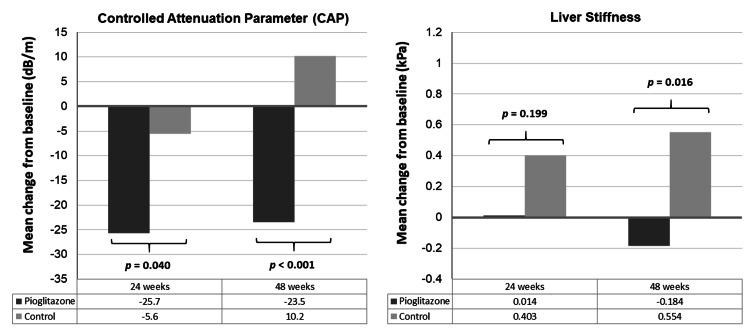
Mean change of CAP and liver stiffness at 24 and 48 weeks from baseline between patients in pioglitazone (black color) and control (grey color) groups

At 24 weeks, the mean change of CAP was -25.7 ± 7.5 dB/m in the pioglitazone group and -5.6 ± 1.8 dB/m in the control group (p = 0.040); the mean change of liver stiffness was 0.014 ± 0.005 kPa in the pioglitazone group and 0.403 ± 0.107 kPa in the control group (p = 0.199). At 48 weeks, the mean change of CAP was -23.5 ± 6.9 dB/m in the pioglitazone group and 10.2 ± 3.5 dB/m in the control group (p < 0.001); the mean change of liver stiffness was -0.184 ± 0.087 kPa in the pioglitazone group and 0.554 ± 0.135 kPa in the control group (p = 0.016) (Figure 1).

**Figure 2:**
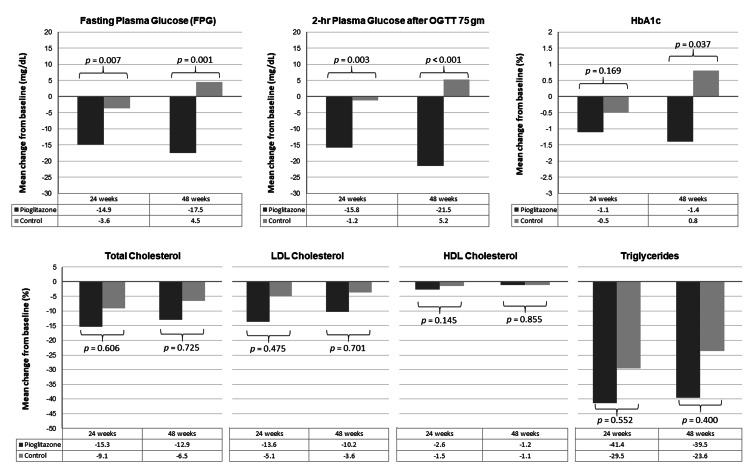
Mean change of FPG, 2h-PG, HbA1c and lipid profiles at 24 and 48 weeks from baseline between patients in pioglitazone (black color) and control (grey color) groups HbA1c: glycosylated hemoglobin; HDL: high-density lipoprotein cholesterol; LDL: low-density lipoprotein cholesterol; OGTT: oral glucose tolerance test; 2h-PG: two-hour plasma glucose

For metabolic parameters, the mean changes of FPG at 24 and 48 weeks were -14.9 ± 3.7 and -17.5 ± 4.2 mg/dL, respectively, in the pioglitazone group and -3.6 ± 0.9 and 4.5 ± 1.3 mg/dL, respectively, in the control group (p = 0.007 at 24 weeks and p = 0.001 at 48 weeks). The mean changes of 2h-PG at 24 and 48 weeks were significantly greater in the pioglitazone group when compared to the control group (Figure 2). Regarding the HbA1c, the mean changes in the pioglitazone group were significantly greater only at 48 weeks. The mean changes of body weight, BMI, lipid profiles, and liver enzymes were not different between the two groups (p > 0.05) at both time points. No serious adverse effects were observed in either group throughout the study. One patient in the pioglitazone group had a 2 kg weight gain in 12 weeks and the body weight was stable at 24 to 48 weeks. There were no patients lost to follow-up during the study.

## Discussion

The results of the present study showed that, in addition to weight control and exercise, medical treatment with pioglitazone in PLHIV with prediabetes and MAFLD was effective in reducing CAP values when compared to placebo. The changes were obvious starting at the 24 weeks follow-up. However, the mean changes in liver stiffness between the two treatment groups were not statistically significant until 48 weeks of treatment. Pioglitazone therapy in these patients also significantly improved the FPG and 2h-PG starting at 24 weeks of treatment, whereas HbA1c was significantly improved at 48 weeks of study.

Currently, none of the medical treatments are approved for MAFLD, in both the general population and PLHIV. Some treatment guidelines suggest that pioglitazone may histologically improve non-alcoholic steatohepatitis in terms of liver steatosis, inflammation, and hepatocytes ballooning in HIV-uninfected patients [23, 25]. In clinical practice, pioglitazone is not commonly prescribed for HIV-uninfected patients with MAFLD. However, the prevalence of MAFLD in PLHIV is substantially higher than that in the general population. Furthermore, the disease more rapidly progresses to steatohepatitis, cirrhosis, and cirrhotic-related complications in PLHIV [10]. Similarly, type 2 diabetes and prediabetes are prevalent in PLHIV [26-27]. Accordingly, determining the treatment that could delay or prevent the progression to type 2 diabetes, reduce insulin resistance, and improve steatohepatitis and liver fibrosis is clinically meaningful in PLHIV with prediabetes and MAFLD. Therefore, determining the effect of pioglitazone in PLHIV and prediabetes who have MAFLD is beneficial for caring for these patients.

This study is the first randomized controlled study that evaluated the efficacy of pioglitazone for the treatment of MAFLD in PLHIV and prediabetes. When compared to the placebo group, more reduction in CAP as early as 24 weeks was demonstrated in the pioglitazone group, and this benefit was persisted at 48 weeks after the treatment. Therefore, pioglitazone could improve liver steatosis. An increase in liver triglycerides content caused by the chronic release of free fatty acids from insulin-resistant dysfunctional adipose tissue is one of the major causes of MAFLD [28]. It is well-established that pioglitazone improves insulin resistance and adipose tissue dysfunction. In addition, pioglitazone also induced significant improvements in most glucose- and lipid-related parameters, including insulin sensitivity, HbA1c level, triglyceride level, and high-density lipoprotein cholesterol level [29].

Correspondingly, we demonstrated significant improvements in FPG, 2h-PG, and HbA1c. However, we did not see an improvement in liver enzymes as in a previous study [30]. The explanation could be that our participants had a lower level of liver enzymes at baseline when compared with that in the previous study. Unexpectedly, the changes in body weight were similar among the two groups; and only one patient in the pioglitazone had a gain of 2-kg in body weight. It is well-known that pioglitazone causes significant weight gain at approximately 2 - 3 kg [28, 30]. Since our participants had a lower BMI at baseline when compared with that in the previous studies (~25 vs. 33 - 34 kg/m^2^), less weight gain would occur after receiving the treatment.

To the best of our knowledge, our report is the first randomized placebo-controlled study to evaluate the efficacy of pioglitazone for the treatment of MAFLD in PLHIV and prediabetes. The strengths of our study are the study design of a randomized placebo-controlled trial in a specific population and the sufficient sample size that enables to demonstrate the efficacy of pioglitazone therapy.

There were some limitations in our study. Although non-invasive tools, TE with CAP and liver ultrasonography, are increasingly used to replace a liver biopsy in patients with MAFLD, we acknowledge this as a limitation of our study because they are not gold-standard tools for the diagnosis of non-alcoholic steatohepatitis. However, a substantial number of clinical studies have shown the accuracy of CAP compared with a liver biopsy for an evaluation of liver steatosis [25]. Since the majority of study patients in both groups were in the F0 - F1 range of liver stiffness, and even though there was a significant reduction, almost all patients were still in the same range. The duration of our study was 48 weeks and we were unable to assess the long-term outcomes, such as cirrhosis and cirrhotic-related complications. Thus, the long-term clinical benefits are still uncertain.

## Conclusions

In conclusion, pioglitazone therapy appears to be safe and significantly reduces CAP and liver stiffness in PLHIV with prediabetes and MAFLD. The change of CAP was obvious starting at the 24-week follow-up, whereas the change in liver stiffness was not significant until the 48-week follow-up. Parameters of glucose metabolism were also improved significantly. However, pioglitazone therapy had no effect on BMI, lipid profiles, and liver enzymes. Further study with a longer study duration is warranted to determine the roles of pioglitazone for clinical use in this population.

## References

[1] (2021). Global HIV & AIDS Statistics — Fact Sheet. http://www.unaids.org/en/resources/fact-sheet.

[2] Smith CJ, Ryom L, Weber R (2014). Trends in underlying causes of death in people with HIV from 1999 to 2011 (D:A:D): a multicohort collaboration. Lancet.

[3] Althoff KN, Gebo KA, Moore RD (2019). Contributions of traditional and HIV-related risk factors on non-AIDS-defining cancer, myocardial infarction, and end-stage liver and renal diseases in adults with HIV in the USA and Canada: a collaboration of cohort studies. Lancet HIV.

[4] Vallet-Pichard A, Mallet V, Pol S (2012). Nonalcoholic fatty liver disease and HIV infection. Semin Liver Dis.

[5] Jericó C, Knobel H, Montero M (2005). Metabolic syndrome among HIV-infected patients: prevalence, characteristics, and related factors. Diabetes Care.

[6] Wand H, Calmy A, Carey DL (2007). Metabolic syndrome, cardiovascular disease and type 2 diabetes mellitus after initiation of antiretroviral therapy in HIV infection. AIDS.

[7] Lake JE, Overton T, Naggie S (2020). Expert panel review on nonalcoholic fatty liver disease in persons with human immunodeficiency virus. Clin Gastroenterol Hepatol.

[8] Younossi ZM, Marchesini G, Pinto-Cortez H, Petta S (2019). Epidemiology of nonalcoholic fatty liver disease and nonalcoholic steatohepatitis: implications for liver transplantation. Transplantation.

[9] Mikolasevic I, Orlic L, Franjic N, Hauser G, Stimac D, Milic S (2016). Transient elastography (FibroScan(®)) with controlled attenuation parameter in the assessment of liver steatosis and fibrosis in patients with nonalcoholic fatty liver disease - where do we stand?. World J Gastroenterol.

[10] Hernaez R, Lazo M, Bonekamp S, Kamel I, Brancati FL, Guallar E, Clark JM (2011). Diagnostic accuracy and reliability of ultrasonography for the detection of fatty liver: a meta-analysis. Hepatology.

[11] Matta B, Lee TH, Patel K (2016). Use of non-invasive testing to stage liver fibrosis in patients with HIV. Curr HIV/AIDS Rep.

[12] Lemoine M, Assoumou L, De Wit S (2019). Diagnostic accuracy of noninvasive markers of steatosis, NASH, and liver fibrosis in HIV-monoinfected individuals at risk of nonalcoholic fatty liver disease (NAFLD): results from the ECHAM study. J Acquir Immune Defic Syndr.

[13] Desouza CV, Shivaswamy V (2010). Pioglitazone in the treatment of type 2 diabetes: safety and efficacy review. Clin Med Insights Endocrinol Diabetes.

[14] Bril F, Kalavalapalli S, Clark VC (2018). Response to pioglitazone in patients with nonalcoholic steatohepatitis with vs without type 2 diabetes. Clin Gastroenterol Hepatol.

[15] Cusi K, Orsak B, Bril F (2016). Long-term pioglitazone treatment for patients with nonalcoholic steatohepatitis and prediabetes or type 2 diabetes mellitus: a randomized trial. Ann Intern Med.

[16] Razavizade M, Jamali R, Arj A, Matini SM, Moraveji A, Taherkhani E (2013). The effect of pioglitazone and metformin on liver function tests, insulin resistance, and liver fat content in nonalcoholic fatty liver disease: a randomized double blinded clinical trial. Hepat Mon.

[17] Aithal GP, Thomas JA, Kaye PV (2008). Randomized, placebo-controlled trial of pioglitazone in nondiabetic subjects with nonalcoholic steatohepatitis. Gastroenterology.

[18] (2021). Addendum 2. Classification and diagnosis of diabetes: standards of medical care in diabetes-2021. Diabetes Care.

[19] American Diabetes Association (2010). Standards of medical care in diabetes--2010. Diabetes Care.

[20] Boursier J, Zarski JP, de Ledinghen V (2013). Determination of reliability criteria for liver stiffness evaluation by transient elastography. Hepatology.

[21] Castera L, Friedrich-Rust M, Loomba R (2019). Noninvasive assessment of liver disease in patients with nonalcoholic fatty liver disease. Gastroenterology.

[22] Myers RP, Pollett A, Kirsch R (2012). Controlled attenuation parameter (CAP): a noninvasive method for the detection of hepatic steatosis based on transient elastography. Liver Int.

[23] Chalasani N, Younossi Z, Lavine JE (2018). The diagnosis and management of nonalcoholic fatty liver disease: practice guidance from the American Association for the Study of Liver Diseases. Hepatology.

[24] Younossi ZM, Loomba R, Rinella ME (2018). Current and future therapeutic regimens for nonalcoholic fatty liver disease and nonalcoholic steatohepatitis. Hepatology.

[25] Francque S, Vonghia L (2019). Pharmacological treatment for non-alcoholic fatty liver disease. Adv Ther.

[26] Tripathi A, Liese AD, Jerrell JM, Zhang J, Rizvi AA, Albrecht H, Duffus WA (2014). Incidence of diabetes mellitus in a population-based cohort of HIV-infected and non-HIV-infected persons: the impact of clinical and therapeutic factors over time. Diabet Med.

[27] Srivanich N, Ngarmukos C, Sungkanuparph S (2010). Prevalence of and risk factors for pre-diabetes in HIV-1-infected patients in Bangkok, Thailand. J Int Assoc Physicians AIDS Care (Chic).

[28] Cusi K (2012). Role of obesity and lipotoxicity in the development of nonalcoholic steatohepatitis: pathophysiology and clinical implications. Gastroenterology.

[29] Chilcott J, Tappenden P, Jones ML, Wight JP (2001). A systematic review of the clinical effectiveness of pioglitazone in the treatment of type 2 diabetes mellitus. Clin Ther.

[30] Belfort R, Harrison SA, Brown K (2006). A placebo-controlled trial of pioglitazone in subjects with nonalcoholic steatohepatitis. N Engl J Med.

